# Development and Validation of a Smartphone Heart Rate Acquisition Application for Health Promotion and Wellness Telehealth Applications

**DOI:** 10.1155/2012/696324

**Published:** 2012-01-05

**Authors:** Mathew J. Gregoski, Martina Mueller, Alexey Vertegel, Aleksey Shaporev, Brenda B. Jackson, Ronja M. Frenzel, Sara M. Sprehn, Frank A. Treiber

**Affiliations:** ^1^South Carolina Center of Economic Excellence, Technology Applications Center for Healthful Lifestyles (TACHL), Medical University of South Carolina, Charleston, SC, USA; ^2^College of Nursing, Medical University of South Carolina, 99 Jonathan Lucas Street, Charleston, SC 29425, USA; ^3^Division of Biostatistics and Epidemiology, Medical University of South Carolina, Charleston, SC 29425, USA; ^4^Department of Bioengineering, Clemson University, Clemson, SC 29634, USA; ^5^College of Medicine, Medical University of South Carolina, 99 Jonathan Lucas Street, Charleston, SC 29425, USA

## Abstract

*Objective*. Current generation smartphones' video camera technologies enable photoplethysmographic (PPG) acquisition and heart rate (HR) measurement. The study objective was to develop an Android application and compare HRs derived from a Motorola Droid to electrocardiograph (ECG) and Nonin 9560BT pulse oximeter readings during various movement-free tasks. *Materials and Methods*. HRs were collected simultaneously from 14 subjects, ages 20 to 58, healthy or with clinical conditions, using the 3 devices during 5-minute periods while at rest, reading aloud under observation, and playing a video game. Correlation between the 3 devices was determined, and Bland-Altman plots for all possible pairs of devices across all conditions assessed agreement. *Results*. Across conditions, all device pairs showed high correlations. Bland-Altman plots further revealed the Droid as a valid measure for HR acquisition. Across all conditions, the Droid compared to ECG, 95% of the data points (differences between devices) fell within the limits of agreement. *Conclusion*. The Android application provides valid HRs at varying levels of movement free mental/perceptual motor exertion. Lack of electrode patches or wireless sensor telemetric straps make it advantageous for use in mobile-cell-phone-delivered health promotion and wellness programs. Further validation is needed to determine its applicability while engaging in physical movement-related activities.

## 1. Introduction

Many cellular phones now possess high-speed data transmission capabilities (e.g., 3G, 4G) and have embedded microprocessors (e.g., Bluetooth, ANT) with the capability to wirelessly connect to external devices. As a result, cell phones offer several advantages over desktop or laptop computers in telemonitoring-related applications such as higher population penetration, increased privacy, lower cost to purchase, easier ability to transport, and overall increased personal convenience of use [1, 2]. Cell phones are widely used in telemonitoring serving as a conduit for receiving biohealth information from portable medical devices (e.g., blood pressure, glucose and pulse oximeter monitors, weight scales) and mobile sensors (e.g., physical activity; accelerometer counts, heart rate, respiration rate, pulse pressure via chest- or armbands, and wireless electrodes). Once cell phones have received the pertinent information, it is microprocessed, encrypted, and the data packets are transferred to some form of localized or web-based server for secondary processing. At the server level, the data packets are organized into a functional database for analyses, integration, and user feedback. The process enables users the ability to easily self-monitor various health parameters and provides important information to healthcare providers facilitating timely healthcare decisions [3–8]. The capture and transfer external biomarker signals from cell phones are not without potential hindrances. Some of the biomarker devices can be fairly expensive to purchase and/or maintain functionality. There is the potential of unexpected loss of wireless sensor connection, increased power depletion upon the cell phone for implementation of the device, and often a burden of responsibility in terms of keeping track of the external detection device. Further, many of these devices require the wearing of multiple sensors, which may be somewhat uncomfortable, and possibly impede movement.

Assessment of electrocardiographic- (ECG-) derived heart rate (HR) in the natural environment is a common component of health and fitness programs. Wireless HR monitors are widely available and provide users with real-time feedback of their HR at rest, during and following physical and/or mental exertion [9, 10]. However, wireless HR monitors often require wearing a telemetric strap around the thoracic region or arm, maintaining vigilance, and making intermittent adjustments of the device to ensure continued proper placement. An alternative approach is through use of a pulse oximeter, a small device that uses photoplethysmography (PPG) to capture blood volume change by illuminating the finger with a light-emitting diode (LED) and measuring the changes in skin illuminated light by transmitting it through a photodiode. Although easy to position on the finger, pulse oximeters are somewhat obtrusive and impractical for intermittent use throughout the day.

The current generation of cellular phones includes video recording capabilities using a photodetector with an accompanying light-emitting diode (LED) light source positioned close to or surrounding the photodetector lens. Verkruysee et al. (2008) showed that the camera photodetector signal, rather than photodiode, enables PPG imaging with improved sensitivity [11]. Jonathan and Leahy [12] recently conducted a single case study and demonstrated that reflection PPG imaging of an individual's finger tip using a consumer grade third-generation cell phone (Nokia model E63) in video mode was able to detect changes in HR from rest to after exercise [12]. However, no attempt was made to determine the validity of this technique.

The purpose of the present study was to determine the validity of an Android-based software program to detect and capture HR measurements as a proof of concept for its use. The system was developed and tested on a Motorola Droid (Motorola, Libertyville, IL) running Android OS 2.2 (Google, Mountain View, CA). The Android program utilizes the original equipment manufacturers (OEM) video hardware and software. HRs derived from this application were compared to those acquired using a four-lead ECG, as well as a pulse oximeter. Subjects were evaluated at rest, while playing a challenging video game and reading aloud.

## 2. Materials and Methods

### 2.1. HR Detection and Processing Software Development Procedures

The application utilizes an imaging acquisition concept similar to that found in commercially available pulse oximeters in which infrared light is used to determine oxygenated and deoxygenated blood based on the blood opacity (i.e., oxygenated is brighter red than deoxygenated which is blue/purple). Variable levels of opacity are illuminated by the camera light and measured by the camera's five-megapixel photodetecting lens, as it passes through the pulsating capillary tissue beneath the surface of the finger. Measurements are tracked overtime and analyzed until a pulsatile signal is properly detected. The first step was to determine if the image captured from the light provided by original equipment manufacturer's (OEM) LED flash and five-megapixel camera could be passed through software image analysis filters to detect the appropriate wavelengths for efficient pulse detection based on variations in opacity. The next step was to determine whether the smartphone could process frame-by-frame image analysis in a timely and consistent manner, in order to detect the small pulsatile color changes. An nv21 YUV planar frame format was used to allow for efficient processing of both light and color intensity changes. Real-time trials were used to determine an optimum settings bundle of frame capture rate (20 fps), image pixel density (176 × 144), focus mode (infinity), and software image analysis filters. The next step was to detect changes of blood flow by comparing values over a series of frames. During this step, points were gathered and graphed to help programmers visually discern changes of blood flow. Throughout this process, the development team compared signal detection by the developed application with a Nonin Onyx II model 9560BT oximeter (Nonin Medical, Plymouth, MN). Initial comparisons allowed assurance of capturing opacity changes representative of accurate pulsatile readings during the recording and graphing of data. A history system aids the detection of HR pulses as well as accumulates the pulses to generate beats per minute HR feedback. Once the appropriate signal-to-noise ratio was established, minor adjustments were made to ensure noise or small changes in the user's finger position did not disrupt the continuous monitoring of HR. Finger movements that resulted in unobtainable HR measurement or finger removal results in vibration-based feedback to the user to reposition the finger on the camera lens. The smartphone HR monitor application directs each step of the process from video capture, video processing and analysis, and feedback to the user in a graphical user interface.

### 2.2. Subjects

The institutional review board approved the study. A convenience sample of 14 adults, aged 18–59, volunteered and participated in the study. The diverse sample of participants consisted of 11 females, 6 non-Hispanic Whites, 3 Hispanic Whites, and 5 Non-Hispanic African-Americans. Following informed written consent, subjects had their heights and weights measured and provided a brief health history of chronic diseases and medication usage. Descriptive characteristics are presented in Table 1.

### 2.3. Evaluation Procedures

**Figure 1:**
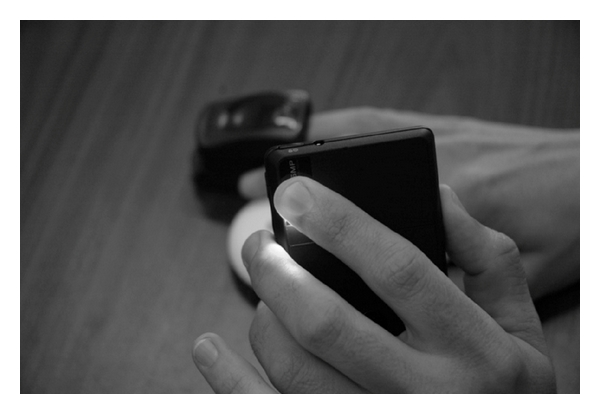
Placement of index finger with Droid device.

Following anthropometric measurements, subjects had two sets of electrodes interfaced with a BioZ thoracic bioimpedance monitor (SonoSite, Bothell, WA) placed 5 cm apart at the level of the xiphoid notch in the midaxillary line and at the angle of the jaw on each side of the neck. The sensors formed four ECG vectors. The BioZ detects these ECG vectors, and the RR intervals are converted into beat-to-beat HRs including a record of the real time of each beat. To match readings provided by the Nonin 9560, the BioZ ECG was set to measure 4 beat rolling averages. Subjects were then seated at a desk, at a comfortable distance (“15” to “20”) from a “15” diagonal laptop computer screen, and the cell phone was held in the left hand with the index finger covering the lens and LED (see Figure 1). All subjects were right handed.

**Figure 2:**
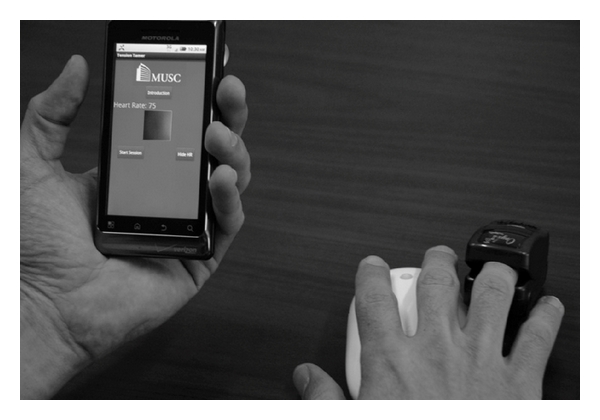
Placement of Droid and Nonin devices during data acquisition.

The left arm was positioned in proximity to the BioZ impedance monitor screen such that both the phone screen and BioZ screen were visible on a video camera. The subject was given a cushioned object to use to comfortably hold his/her left arm in position. The Nonin pulse oximeter was placed on the subject's right-hand ring finger (see Figure 2). Data from this device were transmitted via Bluetooth to a laptop where time-stamped HR data were recorded every second during the 3 five-minute evaluation conditions. A Panasonic model VDR-M50 video camera was used to record the BioZ and Motorola Droid display screens throughout each condition. Video was captured to computer file by PowerDirector (CyberLink, Santa Clara, CA). Files were then reviewed frame by frame and displayed HRs recorded.

The subject was then directed to sit quietly, without crossing his/her legs for 5 minutes. Next, the subject was given printouts of recent local newspaper articles to read aloud for 5 minutes. During these evaluations two research assistants were present observing the presentation. Finally, the 5-minute Atari video game “Break Out” was presented via computer emulator on the “15” diagonal laptop screen. This game has been used in a number of laboratory stress cardiovascular reactivity evaluation studies and has shown to elicit significant increases in HR, and other hemodynamic functions as well as being an independent predictor of future blood pressure levels [13, 14]. The video game involves knocking bricks out of a brick wall. A ball drops from the top of the wall and one tries to bounce it back to knock out bricks via timing positioning of a small paddle under where the ball is directed. The participant controlled placement of the paddle by slight sideways movements of a computer mouse. The slight movements did not interfere with signal acquisition and transmission on the Nonin 9560BT. After completion of the three conditions, each subject's data from the three devices were date stamp matched. Video recordings of the Droid cell phone screen, which displayed 4 beat rolling average HRs, were synchronized with data recorded from the other devices. The HR data from each device were averaged for each minute such that there were 5 separate average HRs for each device per each condition.

### 2.4. Data Analyses

The ECG-derived average HRs per minute from the BioZ bioimpedance cardiograph system using the 4 beat rolling average served as the criterion measure. HR values recorded from the cell phone and the Nonin pulse oximeter were averaged and compared to the ECG at their respective time periods. Pearson correlation coefficients (*r*) and standard errors of estimate (SEE) were calculated. As devices are expected to be highly correlated measuring the same signal agreement of the 3 devices was assessed for each activity using Bland Altman plots for repeated measures with 95% limits of agreement [15].

## 3. Results

All participants completed the testing procedures.  Table 2 portrays the HRs obtained via the different devices and during the three conditions.  Table 3 displays the correlation coefficients and standard errors of estimate (SEE) for the different conditions. As expected, mean HRs were slightly increased for the reading aloud and video game challenge conditions, compared to the resting sitting condition.

Previous research has suggested that HR monitors are valid if the correlation coefficients are ≥.90 and the SEEs are ≤5 beats per minute (bpm) [16]. The HRs obtained from the Motorola Droid cell phone were highly correlated with those from the BioZ ECG (*r* ≥ .99), and the SEEs calculated between the Droid and BioZ ECG were ≤1 bpm during all conditions. There was also high correlation between the Motorola Droid and the Nonin 9560BT during all conditions (*rs* ≥ .99, SEE ≤ 2.09 bpm). However, high correlations between multiple monitors do not necessarily equate to adequate agreement, as data from two HR devices can align on any linear plane and will result in high correlation coefficients.

**Figure 3:**
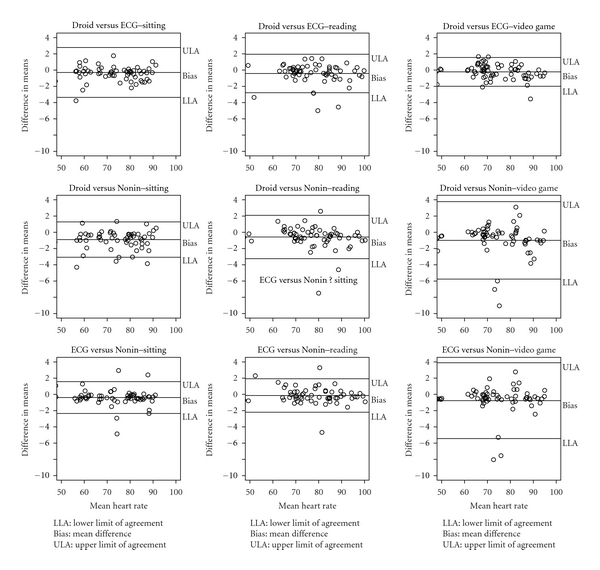
Bland-Altman plots comparing agreement between Droid, Nonin, and ECG at three activities.

To determine if adequate agreement existed across devices, Bland Altman plots with 95% limits of agreement were developed across all conditions between all possible pairs of devices (Figure 3). All pairings of devices showed good agreement, that is, ≥95 percent of data points (differences between measurement methods) falling between the limits of agreement, except for ECG versus Nonin during reading which had slightly lower agreement with 94%. The width of the 95% limits of agreement ranged from 3.9 bpm for the ECG versus Droid during videogame and ECG versus Nonin during sitting at rest to 9.5 bpm for both ECG and Droid versus Nonin during the video game challenge.

## 4. Discussion

The present findings are based on a limited-size group but the results provide preliminary evidence that our Android software program using the Motorola Droid OEM video imaging system provided valid measurement of HRs across three different tasks which varied in HR intensity. This easy-to-use application has significant potential for use in collecting intermittent HR measurements during environmental activities without having to attach spot electrodes, wiring harness or chest or arm straps, and/or wear wristwatch like receiver devices. The device is able to accurately detect ranges of HR across individuals, comparable to those produced by activities of light intensity.

HR monitoring has played an integral role in the field of health promotion and disease prevention. Electrocardiography (ECG) is the gold standard for beat-to-beat heart rate measurement. However, financial costs of ECG holter monitoring equipment and cumbersome attachment of multiple patch electrodes with wire connections to the device make this methodology impractical for most individuals interested in monitoring their HRs in the natural environment. As described earlier, a number of user friendly and affordable HR monitors are widely available which have been validated at rest and during various levels and types of physical activity [16–19]. However, these devices are still somewhat problematic in terms of requiring the wearing of a telemetric strap, ensuring shifting capable of measurement interference does not occur, and maintaining signal and receiver connections between the telemetric strap and watch interface.

Recently, Lee and Gorelick (2011) validated an HR wristwatch monitor, which does not require wearing a telemetric strap nor patch electrodes [20]. The back of the watch casing serves as one electrode (for wrist conductivity), while two front casing electrodes are located above and below the wristwatch display (for finger conductivity). The watch displays the HR when the fingers of the opposite hand are placed on the electrodes. The device was shown to provide valid HR readings at rest and during treadmill walking and light jogging. It tended to lose ability to acquire HRs at higher treadmill speeds. Although an improvement over chest straps and electrode attachments, one still has to purchase and wear an external device. The Android HR acquisition application circumvents this for those with generation 3 smartphones with similar OEM equipment to the device we tested (i.e., ≥5 megapixel cameras with ≥4x zoom, autofocus, DVD quality (720 × 480 resolution) up to 24 fps capture; up to 30 fps playback and dual LED). In order to fully ensure the OEM equipment is provided with other brands/makes of Android-based OS smartphones, additional validation is required.

Compared to the ECG readings, the Motorola Droid smartphone acquired valid HR at rest and during several mildly stressful situations intended to increase HR levels without physical exertion (i.e., oral reading in front of others, challenging video game). In addition, the smart phone application provided accuracy similar to the FDA-approved (K081285, 2008) and ISO 9919 compliant, Nonin Onyx II model 9560BT ambulatory finger pulse oximeter. The Nonin 9560BT was also shown to provide accurate measurements compared to the ECG. The Nonin 9560BT provides the user with ongoing feedback of HR changes, as well as oxygen saturation levels, on the device's display screen. The biodata can also be transmitted via Bluetooth to an external computer or Bluetooth-enabled smartphone platform in which ECG wave patterns are displayed in real time and data charted. Our Android smartphone HR acquisition application operates in a similar manner with regard to processing of signals and provision of real-time HR changes on the cell phone screen. In addition, the Android software program is capable of providing the user with feedback charts depicting average HR per minute across continuous periods of time from previous HR recording sessions stored on a secure localized server.

Although the findings are promising, there are a few limitations of the study that warrant discussion. First, the main goal of the study was to demonstrate proof of concept for its use. The methods we used for validation are appropriate for this preliminary study. However, more detailed analysis of validity using both our self-developed Android application and the ECG monitor is warranted. Future studies using a larger sampler in conjunction with a blind source separation analyses via principal component analysis (PCA) and independent component analyses (ICA) would further confer validation from a signal analysis perspective [21]. Second, data were acquired while subjects were stationary. This was done intentionally, as the initial purpose of this proof-of-concept study was to use HR acquisition software to facilitate HR monitoring of individuals while stationary and practicing smart-phone-delivered breathing meditation. It is unclear whether the device is capable of capturing HR while jogging and running, which require significant upper body movement including that of the arms and hands. Although only one hand is involved with the Droid device, as opposed to both hands with Lee and Gorelick's [20] wristwatch monitor, further testing is required to determine whether individuals can maintain proper position of their finger on the back of the phone while moving and, if so, can the software detect accurate HR signals. Activities such as slow walking and/or stationary bicycling may be more feasible conditions for using the Android smartphone HR detection software.

In addition, although a wide age range of individuals including both sexes and multiple ethnicities were evaluated, no children were involved in the study. It is possible that children's smaller diameter fingertips may result in less consistent detection of pulsatile flow changes on the camera lens. We are currently using the Android HR acquisition application software as part of a smartphone-delivered breathing meditation study involving prehypertensive 6th and 7th grade girls and adults. We are not experiencing any problems with HR acquisition and processing in the girls or the adults. However, as noted above, further evaluation is needed to determine whether the Android software and smartphone are useful in acquiring HRs while engaging in various levels of physical exertion. It may be the case that among children, with less developed running gates, the combined task of holding a smartphone with a finger over the photodetector lens proves challenging.

In conclusion, the results indicate that the Android HR acquisition software embedded in a Motorola Droid smartphone provides valid measurements of HR while at rest and when engaging in mildly stressful motion-free perceptual motor/cognitive activities. This software appears useful for health promotion preventive medicine programs (e.g., stress management programs; monitoring of heart rate pre- and postphysical exertion) in which individuals are interested in evaluating HRs in the natural environment and storing the data without having to purchase an HR monitoring system with accompanying telemetric strap, spot electrodes, and/or wristwatch. The software application and smartphone video camera may be capable of detecting HRs while engaged in walking, possibly jogging, as long as consistent fingertip connection is made with the LED and photodetector lens. However, whether such signal detection is feasible, especially with higher-intensity movement, is unknown.

## Figures and Tables

**Table 1:** Descriptive Characteristics of Sample.

Sex	Race/Ethnicity	Age	Weight (Ib)	Height (in)	Chronic Disease/Medication(s)
F	NHW	49	160	67.5	
M	NHW	57	188	73	EH, HC; Zocor, Propanol
M	NHW	33	162.6	68.25	Adderall
F	NHW	20	116.1	63	
F	NHW	22	120	63	
F	NHW	59	144.4	63.75	
F	NHAA	43	320.2	61.25	
M	HW	32	177	73.25	HC, statin
F	NHAA	22	185	60	
F	HW	47	260.6	65.25	EH;
F	HW	18	145.4	65.25	
F	NHAA	58	182	62.75	
F	NHAA	32	178.2	65.25	
F	NHAA	42	118.5	62.5	
M/F 3/11	NHW/NHAA/HW 6/5/3	38.1	175.6	65.3	Avg
		14.6	53.4	3.9	Stddev

F: female; M: male; NHW: non-Hispanic White; NHAA: non-Hispanic African American; HW: hispanic White; EH: essential hypertension; HC: hypercholesterolemia.

**Table 2:** Heart rate means and SD across conditions and devices.

Device	Sitting	Reading	Video game	All conditions (average)
BioZ-ECG (bpm)	72.47 ± 12.89	77.39 ± 12.65	74.29 ± 11.93	74.72 ± 12.34
Droid (bpm)	71.94 ± 12.98	76.78 ± 12.51	74.10 ± 11.78	74.27 ± 12.27
Nonin (bpm)	72.86 ± 13.01	77.36 ± 12.79	75.09 ± 11.87	75.10 ± 12.73

**Table 3:** Correlations and SEE for the different conditions.

Condition		ECG versus Droid	Droid versus Nonin	ECG versus Nonin
Sitting	*r* SEE	.99 .59	.99 .47	.99 .25

Reading	*r* SEE	.99 .94	.99 .58	.99 .80

Video game	*r* SEE	.99 .66	.99 2.09	.99 2.04
